# Comparative efficacy and procedural outcomes of pulsed field ablation vs. thermal ablation for paroxysmal atrial fibrillation: a systematic review and meta-analysis of randomized trials

**DOI:** 10.1093/ehjopen/oeaf092

**Published:** 2025-08-04

**Authors:** Satyam Krishan, Taha Zaka Ur Rehman, Siddharth Agarwal, Zain Ul Abideen Asad

**Affiliations:** Department of Medicine, Cardiovascular Section, University of Oklahoma Health Sciences Center, 800 Stanton L. Young Blvd, AAT 5400, Oklahoma City, OK 73104, USA; Department of Medicine, Cardiovascular Section, University of Oklahoma Health Sciences Center, 800 Stanton L. Young Blvd, AAT 5400, Oklahoma City, OK 73104, USA; Department of Cardiovascular Medicine, Mayo Clinic, 200 1st Street SW, Rochester, MN 55905, USA; Department of Medicine, Cardiovascular Section, University of Oklahoma Health Sciences Center, 800 Stanton L. Young Blvd, AAT 5400, Oklahoma City, OK 73104, USA

## Abstract

**Graphical Abstract:**
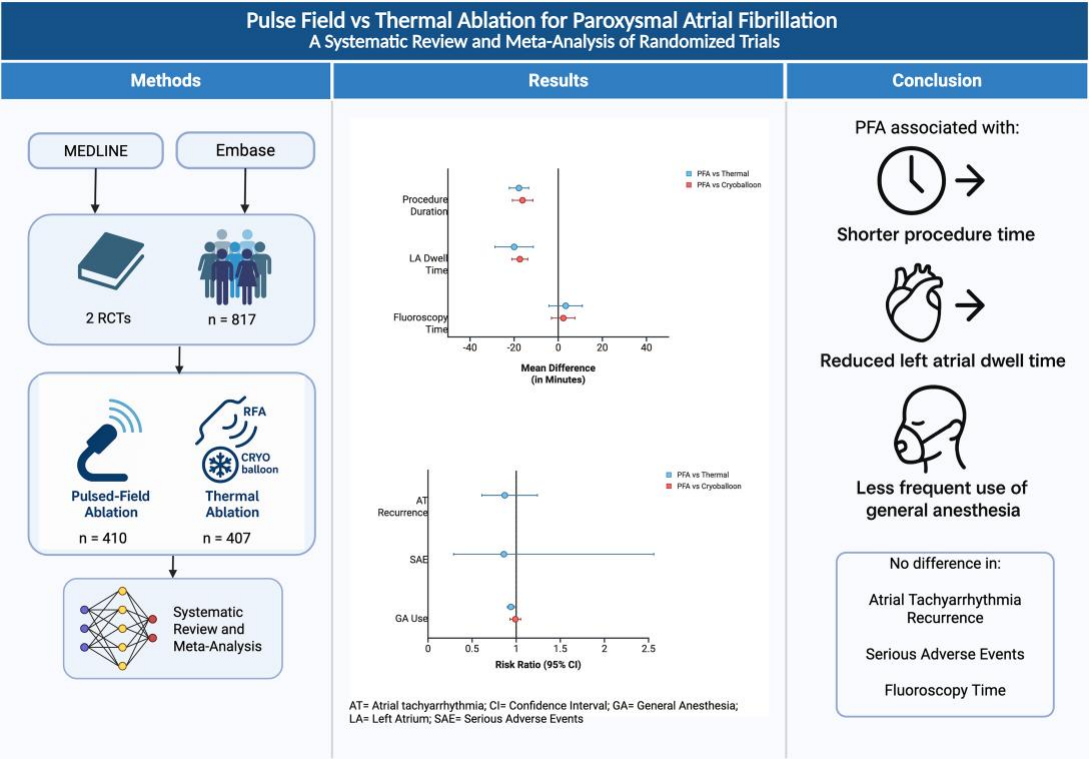

Catheter ablation is an effective treatment for paroxysmal atrial fibrillation (AF) and has conventionally used thermal energy (radiofrequency or cryoballoon) to electrically isolate pulmonary veins which are common triggers for AF.^1^ Pulsed field ablation (PFA) is a novel, non-thermal ablation modality for pulmonary vein isolation, with the potential to selectively target myocardial tissue while minimizing collateral damage to adjacent structures.^2^ While early studies have demonstrated promising safety and efficacy, prior meta-analyses had largely relied on pooled analyses of single-arm observational studies, limiting the strength of comparative conclusions.^3,4^ To date, only two randomized controlled trials (RCTs) have directly compared PFA to conventional thermal ablation strategies, with one of these trials published only recently.^5,6^ Given the growing clinical interest in PFA and the emergence of more randomized data, we conducted a systematic review and meta-analysis of RCTs to directly compare the clinical and procedural outcomes of PFA vs. thermal ablation in patients undergoing catheter ablation for AF.

A systematic search of MEDLINE and EMBASE databases was conducted through April 2025 in accordance with PRISMA guidelines. We used the following medical subject headings: ‘atrial fibrillation’, ‘pulsed field ablation’, ‘radiofrequency ablation’, ‘cryoballoon ablation’, ‘thermal ablation’, and ‘catheter ablation’. Studies were included if they were randomized trials comparing PFA to thermal ablation. Outcomes of interest included atrial tachyarrhythmia recurrence, anti-arrhythmic drug initiation, repeat ablation and cardioversion at 12 months, and procedural parameters including procedure time, left atrial (LA) dwell time, fluoroscopy time, general anaesthesia (GA) use, and peri-procedural serious adverse events (SAEs). Serious adverse events were defined as a composite of cardiac tamponade, phrenic nerve palsy persisting beyond the procedure, major vascular complications, stroke or transient ischaemic attack, atrioesophageal fistula, or death. Data were extracted on an intention-to-treat basis. Risk ratios (RRs) with 95% confidence intervals (CIs) were calculated using the Mantel–Haenszel random effects model for dichotomous outcomes; mean differences (MD) with 95% CIs were calculated for continuous outcomes. Heterogeneity was assessed using the *I*² statistic. A two-tailed *P* < 0.05 was considered statistically significant. Statistical analyses were performed using Review Manager (Version 5.4, Copenhagen: The Nordic Cochrane Centre, The Cochrane Collaboration, 2014). Forest Plots were generated in RevMan. Graphical abstract was created using BioRender.com and licensed for publication under BioRender's Publication License for Open Access.

The search identified 1193 articles of which 549 duplicates were removed and 644 unique records were screened. After the exclusion of review articles, single-arm studies, retrospective studies, and conference abstracts, 20 studies were selected for full-text review. Two RCTs, comprising a total of 817 patients (PFA = 410; thermal ablation = 407), met the inclusion criteria for quantitative synthesis. Compared with thermal ablation, PFA was associated with significantly shorter procedure duration (MD −17.8 min; 95% CI −22.2 to −13.4; *P* < 0.001), reduced LA dwell time (MD −20.0 min; 95% CI −28.7 to −11.3; *P* < 0.001), and lower utilization of GA (RR 0.94; 95% CI 0.90–0.98; *P* = 0.005). No statistically significant differences were observed between groups for procedural fluoroscopy time, atrial tachyarrhythmia recurrence, anti-arrhythmic drug use, repeat ablation, or cardioversion at 12-month follow-up. Rates of peri-procedural SAEs were also comparable between groups. In a subgroup analysis comparing PFA with cryoballoon ablation (including cryoballoon arm from ADVENT and SINGLE SHOT CHAMPION), PFA remained associated with significantly shorter procedure time (MD −16.2 min; 95% CI −20.8 to −11.5; *P* < 0.001) and LA dwell time (MD −17.4 min; 95% CI −21.0 to −13.8; *P* < 0.001). No significant differences were observed between PFA and cryoballoon ablation with respect to fluoroscopy time or GA utilization. Results are shown in *Figure 1*.

**Figure 1:**
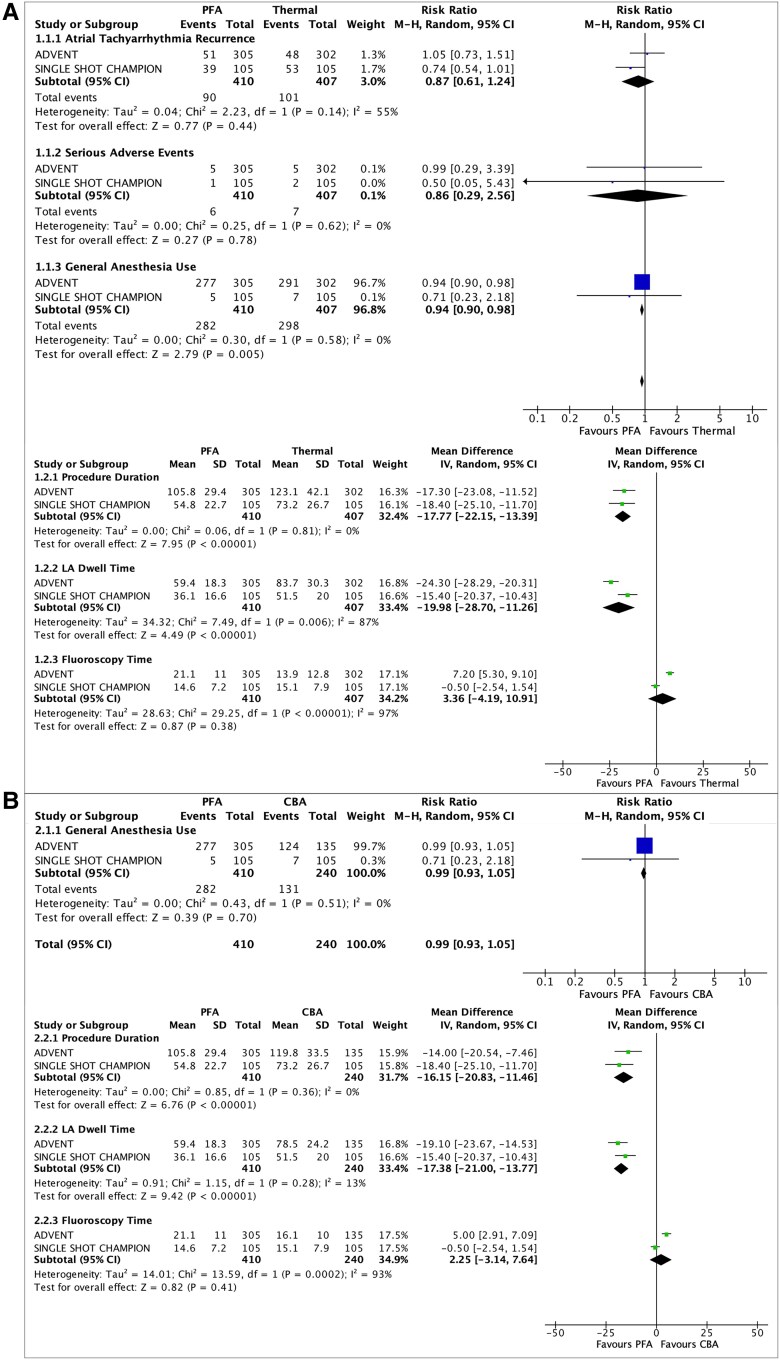
Forest plots comparing pulsed field ablation and thermal ablation. Forest plots summarizing outcomes from randomized controlled trials comparing pulsed field ablation and thermal ablation in patients undergoing catheter ablation for atrial fibrillation. (*A*) Comparison between pulsed field ablation and thermal ablation (radiofrequency or cryoballoon) for atrial tachyarrhythmia recurrence, peri-procedural serious adverse events, general anaesthesia use, and procedural outcomes, including procedure time, left atrial dwell time, and fluoroscopy time. (*B*) Subgroup analysis comparing pulsed field ablation with cryoballoon ablation for general anaesthesia use, procedure time, left atrial dwell time, and fluoroscopy time. Effect sizes are shown as mean differences for continuous outcomes and risk ratios for dichotomous outcomes, each with 95% confidence intervals.

In this meta-analysis of RCTs, PFA showed several procedural advantages over thermal ablation, including significantly shorter overall procedure times, reduced LA dwell time, and less frequent use of GA. The lower need for GA likely reflects the efficient nature of PFA, which allows for rapid, single-pass pulmonary vein isolation with minimal catheter manipulation. However, this result should be interpreted cautiously, given the variability in anaesthesia protocols between trials. In ADVENT, sedation type was left to operator discretion, while in SINGLE SHOT CHAMPION, GA was reserved for high-risk patients.^5^ Furthermore, while cryoballoon ablation is often performed under moderate sedation, RF and PFA typically involve deep sedation or GA, depending on operator experience, catheter design, and institutional protocols. These differences may have influenced anaesthesia-related outcomes in our analysis.

Notably, no significant difference in fluoroscopy time was observed between PFA and thermal ablation overall. In ADVENT, PFA had longer fluoroscopy times compared to cryoballoon and RF, whereas in SINGLE SHOT CHAMPION, fluoroscopy times between PFA and cryoballoon were similar. Importantly, while fluoroscopy durations were similar, SINGLE SHOT CHAMPION demonstrated significantly lower radiation dose and contrast volume with PFA, likely due to reduced reliance on cineangiography during PFA procedures. As future technologies integrate PFA systems with electro-anatomical mapping, both fluoroscopy use and radiation exposure may decline further.^6^

Efficacy outcomes including atrial tachyarrhythmia recurrence, anti-arrhythmic drug use, repeat ablation, and cardioversion at 12 months were similar between PFA and thermal ablation. However, in the SINGLE SHOT CHAMPION trial, PFA was not only non-inferior but also demonstrated borderline superiority to cryoballoon ablation with respect to arrhythmia recurrence.^6^ This may suggest that as techniques mature and technology advances, PFA may offer not only procedural but also clinical advantages. Importantly, there were no significant differences in peri-procedural SAEs, supporting the safety profile of PFA in contemporary clinical practice.^7^

The results of this study should be interpreted with caution in light of the following limitations. First, our meta-analysis is based on aggregate data extracted from original studies, and therefore, patient-level data could not be assessed. Additionally, heterogeneity in patient characteristics and procedural protocols (particularly sedation, fluoroscopy techniques, and operator experience) may have influenced outcomes. Furthermore, only two RCTs were included, limiting the ability to assess publication bias or perform meaningful subgroup analyses. Both trials focused on patients with paroxysmal AF and relatively healthy atrial substrate, so these results may not apply to more complex AF populations. Lastly, with follow-up limited to 12 months, we are unable to comment on long-term efficacy or lesion durability.

To our knowledge, this is the first and only meta-analysis of RCTs directly comparing PFA with thermal ablation for AF. While earlier studies and meta-analyses provided important preliminary insights, they were inherently limited by confounding, selection bias, and heterogeneity in study design due to their reliance on observational or single-arm data. In contrast, the present analysis synthesizes data exclusively from RCTs, providing more robust and precise summary estimates of both procedural efficiency and clinical outcomes at 12 months. As the adoption of PFA continues to expand and additional randomized data become available, future analyses will be better positioned to assess long-term outcomes, lesion durability, and cost-effectiveness. In the interim, our findings support the growing role of PFA as a safe, efficient, and potentially superior alternative to conventional ablation strategies.

## Data Availability

This study utilized data extracted from previously published RCTs. All data underlying the findings are available in the original published articles, which are cited in the manuscript. No new data were generated for this analysis.

## Lead author biography

Dr Satyam Krishan is a cardiology fellow at the University of Oklahoma Health Sciences Center and a member of the American College of Cardiology’s Fellow in Training Section Editorial Board. His academic interests include atrial fibrillation, catheter ablation technologies, and the intersection of cancer and cardiovascular disease. Dr Krishan is actively involved in outcomes research and meta-analyses focused on procedural innovations, comparative effectiveness, and arrhythmia management in both general and oncology populations. He is also pursuing a master’s degree in clinical and translational science with the goal of developing a career as an academic cardiac electrophysiologist.
